# Clinical Factors Implicated in Antibiotic Resistance in *Helicobacter pylori* Patients

**DOI:** 10.3390/microorganisms10020322

**Published:** 2022-01-29

**Authors:** Brian White, Maria Winte, Joshua DeSipio, Sangita Phadtare

**Affiliations:** 1 Department of Biomedical Sciences, Cooper Medical School of Rowan University, Camden, NJ 08103, USA; whiteb64@rowan.edu (B.W.); desipio-joshua@cooperHealth.edu (J.D.); 2 Department of Internal Medicine, Thomas Jefferson University Hospital, Philadelphia, PA 19107, USA; maria.winte@jefferson.edu; 3 Department of Gastroenterology, Cooper University Hospital, Camden, NJ 08103, USA

**Keywords:** *H. pylori*, antibiotic resistance, treatment failure, clinical factors

## Abstract

*Helicobacter pylori* is a common gastric pathogen associated with multiple clinical syndromes, including cancer. Eradication rates of *H. pylori* remain suboptimal despite the progress made in the past few decades in improving treatment strategies. The low eradication rates are mainly driven by antibiotic resistance of *H. pylori*. Non-invasive molecular testing to identify patients with antibiotic-resistant *H. pylori* represents a promising therapeutic avenue, however this technology currently remains limited by availability, costs, and lack of robust validation. Moreover, there is insufficient evidence to demonstrate that resistance-testing-based treatment approaches are superior to appropriately designed empiric strategies. Consensus guidelines recommend use of proven locally effective regimens; however, eradication data are inconsistently generated in several regions of the world. In this review, we describe several clinical factors associated with increased rates of antibiotic resistant *H. pylori*, including history of previous antibiotic exposure, increasing age, female gender, ethnicity/race, extent of alcohol use, and non-ulcer dyspepsia. Assessment of these factors may aid the clinician in choosing the most appropriate empiric treatment strategy for each patient. Future study should aim to identify locally effective therapies and further explore the clinical factors associated with antibiotic resistance.

## 1. Introduction

*Helicobacter pylori* is a ubiquitous microorganism infecting an estimated 50% of adults worldwide [1]. The bacterium utilizes a number of virulence factors to penetrate gastric epithelia, establish infection, and survive in the acidic environment, including blood group antigen-binding adhesion (BabA), cytotoxin-associated gene product (CagA), and neutrophil-activating protein A (NepA) [2]. Although infection is often asymptomatic, it is also associated with several clinical syndromes, including chronic gastritis, peptic ulcer disease, non-ulcer dyspepsia, adenocarcinoma, and mucosa-associated lymphoid tissue (MALT) lymphoma. *H. pylori* is considered a group 1 carcinogen, and its eradication has been shown to reduce the incidence of gastric cancer [3]. Despite constant diligence in improving treatment strategies for *H. pylori* infection, eradication rates remain suboptimal. Antibiotic resistance is the largest driver of eradication failure, despite the use of multidrug regimens. For years, clarithromycin-based triple therapy was considered first-line therapy, but the increasing global prevalence of resistance to multiple agents including clarithromycin has diminished their effectiveness, prompting clinicians to find other strategies to improve treatment success [4]. Encouraging recent data suggest the potential of new, nontraditional therapeutic approaches such as plant-derived polyphenols, including those found in the extract of the Qing Guo fruit [5,6]. Vaccines targeting some of the virulence factors described above have theoretical promise but are still at very early stages of development [7]. Despite the interest these strategies have garnered for future research, antibiotic-based regimens remain the most critical treatment for *H. pylori* at this time. Nevertheless, identifying which antibiotic regimen is most likely to succeed for each patient while also attending to the principles of antibiotic stewardship, i.e., minimizing the harms of antibiotic overuse, has proven ever-more challenging.

Culture-based antibiotic susceptibility testing may offer the most reliable prediction of treatment success. However, cost, time-investment, and dependence on gastric biopsy render this an inappropriate choice for guiding first or second-line therapy [8]. Genotypic methods for detection of antibiotic resistance from biopsy specimens have shown over 95% sensitivity and specificity for identifying clarithromycin and quinolone resistance but their applicability is limited by similar concerns about cost and invasiveness [9]. Non-invasive, stool-based genotypic resistance testing methods have been proposed for guiding therapy [10]. These strategies employ polymerase chain reaction (PCR)-based assays to detect mutations in the *H. pylori 23S rRNA* and *gyrA* genes associated with clarithromycin and fluoroquinolone resistance, respectively. Despite their potential, these methods are limited by concerns about sensitivity and the number of sequences they can target that will be useful for predicting resistance to antibiotics [11,12,13]. Moreover, there is currently insufficient evidence to suggest that genotypic susceptibility-guided treatment strategies are more effective than empiric therapies [14]. Next generation sequencing (NGS)-based strategies to detect a broad array of *H. pylori* mutations associated with antibiotic resistance hold promise. A recent study of one NGS platform for detection of genes conferring resistance to commonly used antibiotics for *H. pylori* found that it could reliably predict resistance to clarithromycin and levofloxacin, but less consistently for metronidazole and amoxicillin [15]. The reliance on sequencing of already-known loci associated with resistance, need for biopsy specimens, and costs currently limit the widespread application of this technology in clinical practice [16]. Authors of current guidelines call for further development of stool-based genotypic testing and comparison of the cost-effectiveness of such susceptibility-guided strategies with empiric therapy, however they stop short of recommending the routine use of susceptibility testing to guide initial treatment at this time [17]. 

Considering the barriers to routine susceptibility testing, authorities contend that empiric treatment strategies should be based on local patterns of resistance and eradication success rates, when these are known. For example, practice guidelines endorse the use of clarithromycin-based triple therapy if the local prevalence of clarithromycin resistance is known to be less than 15% and the patient has no previous macrolide exposure [18]. In the same vein, a local eradication rate greater than 85% is cited as a threshold for efficacy and sufficient evidence for an agent’s empiric use locally [19]. Despite the intuitive benefit of such strategies, there are multiple challenges to the identification of locally effective therapies. Infrequent use of susceptibility testing and inconsistent completion of follow-up eradication testing inhibit the generation of reliable local resistance and efficacy data. Moreover, marked variation between regions and from year-to-year obscures generalization of what data exists. To this point, there has been insufficient surveillance data to consistently identify locally effective regimens [20]. Notably, data on antibiotic resistance among *H. pylori* strains in North America remain particularly scarce [21]. When local antibiotic resistance patterns are unknown, guidelines recommend bismuth-based or non-bismuth-based quadruple therapies as empiric first-line regimens [17]. Nevertheless, cure rates with these regimens are imperfect, with a recent international network meta-analysis of randomized trials finding 81.3% and 84.3% success rates of bismuth and non-bismuth-based quadruple therapies, respectively [22]. In addition to antibiotic resistance, inconvenient four times per day dosing and bothersome side effects may inhibit the effectiveness of these regimens vis-à-vis decreased adherence. Levofloxacin-based regimens showed similar success rates in the network meta-analysis (83.8%), but concerns about increasing resistance and adverse effects preclude these regimens from use as first-line agents [22]. Although vonoprazan-based triple therapies performed well with a 91.4% success rate, authors expressed concern about the decreasing efficacy of this regimen in the face of increasing clarithromycin resistance in Japan [22]. Vonoprazan is currently being considered for regulatory approval in the USA. 

The limited availability of resistance testing and shortcomings of current empiric strategies highlight the need for a new approach to *H. pylori* therapy. While the acquisition of accurate data regarding local resistance patterns remains challenging, identification of individual clinical factors associated with resistance for each patient could help guide selection of the optimal empiric treatment to make it more successful, timely, and cost-efficient. Indeed, data from a study by Liou et al. [23] suggested that properly designed empiric therapy based on clinical risk factors may be as effective as genotypic resistance-based therapy for *H. pylori* eradication. In our recent study, via extensive literature search, we compiled various factors that may contribute to the antibiotic resistance of *H pylori*. We then analyzed data from more than two thousand *H pylori* patients with respect to several of these factors to create a model to predict antibiotic sensitivity/resistance among a subset of patients. Analysis of patient stool samples for known mutations leading to clarithromycin resistance in *H. pylori* allowed us to determine the accuracy of these predictions to be 90%. A median household income of less than $54,000, past *H pylori* infection, previous use of antibiotics such as clarithromycin, cephalexin etc. for any infection decreased the chance of eradication, while past use of PPI, metronidazole and tetracycline increased the chances of eradication. These results suggest that the clinical history may obviate the need for laboratory testing in predicting resistance [24]. Here we review clinical factors associated with antibiotic resistance that may provide avenues for research to predict which patients are at increased risk of treatment failure and subsequently guide the selection of successful and cost-effective personalized empiric therapy.

**Figure 1:**
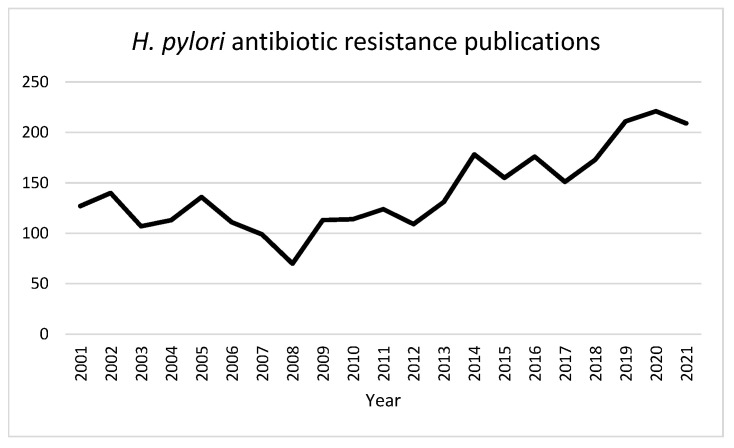
PubMed-indexed publications identified *via* keyword search “*H. pylori*” AND “antibiotic resistance” included per year from 2001–2021.

Meyer et al. [25] carried out meta-analysis of 20 US trials conducted between 1993 and 1999 that tested *H. pylori* isolates from patients with active or inactive peptic ulcer disease. They identified multiple risk factors for resistance to clarithromycin and metronidazole, including geographic region, older age, female sex, inactive ulcer disease, and Asian ethnicity. In light of increasing rates of antibiotic resistance, more prevalent use of fluoroquinolone and other antibiotics, and recognition of *H. pylori* infection in patients without documented ulcer disease, we set out to conduct a more current review of risk factors for resistance. In our review, studies were identified via electronic search of the PubMed database using a mix of key terms including “*H. pylori*”, “*Helicobacter*”, “*Helicobacter pylori*”, “antibiotic resistance”, “age”, “gender”, “sex”, “race”, “ethnicity”, and “alcohol”. Included studies reported rates of *H. pylori* resistance to at least one antibiotic as a function of one or more baseline demographic or clinical characteristics. Outcomes were reported as absolute risks, relative risks (RR), odds ratios (OR), or hazard ratios (HR). Figure 1 shows trend in studies discussing antibiotic resistance of *H. pylori* in the past 20 years. As seen, there has been an increase in the number of studies, especially in the last decade, emphasizing the global urgent need for resolution of this issue.

## 2. Clinical Factors Implicated in Antibiotic Resistance

**Figure 2:**
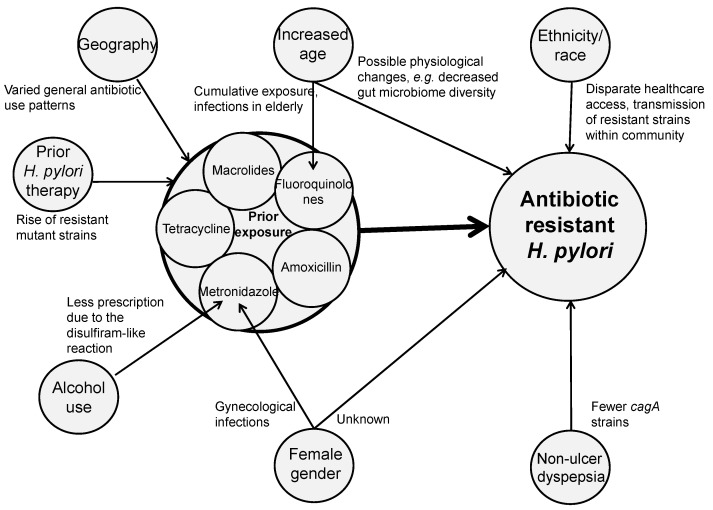
Clinical factors implicated in the development of antibiotic-resistant *H. pylori*. Arrows connect clinical factors to proposed, theoretical mechanisms by which they contribute to antibiotic resistance among *H. pylori* strains.

We identified several clinical factors associated with antibiotic-resistant *H. pylori*. Figure 2 highlights these clinical factors and describes proposed mechanisms by which they might contribute to the development of antibiotic resistance. Intuitively, a major risk factor associated with antibiotic resistance and eradication failure is a patient’s history of prior antibiotic exposure [26,27,28]. Other factors associated with increased rates of antibiotic resistance include geographic location, older age, female gender/sex, ethnicity/race, extent of alcohol use and the type of gastrointestinal pathology (non-ulcer dyspepsia vs. peptic ulcer) [29,30,31,32,33,34]. Rates of antibiotic resistance vary by geographic location, which may reflect regional patterns of community antibiotic use as well as patterns of spread and other healthcare systems-level factors; we do not focus on the impact of this variable in our review [4,30]. Instead, we devote our attention to patient-level factors that vary within a community and may inform the selection of personalized empiric therapy. As shown in Figure 2, most of these factors are likely to contribute to the development of antibiotic resistance and eradication failure vis-à-vis prior exposure to particular antibiotics used in *H. pylori* treatment regimens; however, some of the factors may also contribute to the development of resistance via independent mechanisms. Below we describe the evidence behind associations between clinical factors and antibiotic resistance, as well as postulated mechanisms for these relationships. For each factor, in addition to the discussion in text, data illustrating the relationships between clinical factors and rates of resistance to three commonly used antibiotics in *H. pylori* therapy, clarithromycin, metronidazole, and fluoroquinolones, are shown in Table 1, Table 2, Table 3 and Table 4.

### 2.1. Prior Antibiotic Exposure

Evidence from in vitro, animal, ecological, and clinical cohort studies demonstrate that prior antibiotic exposure is strongly associated with subsequent antibiotic resistance. Serial passage of *H. pylori* on agar media containing ciprofloxacin, metronidazole, erythromycin, and tobramycin yielded strains resistant to these agents [35]. Jenks et al. [36] showed that metronidazole pre-treatment of mice harboring the metronidazole-sensitive *H. pylori* SS1 strain induced the development of metronidazole resistance and significantly lower eradication rates with a metronidazole-containing triple therapy. In a prospective analysis of 2204 patients with *H. pylori* across 18 European countries, Megraud et al. [30] explored the relationships between rates of primary antibiotic resistance and yearly outpatient antibiotic use at the population level, expressed in defined daily doses per 1000 inhabitants per day. They found 17.5%, 14.1%, and 34.9% of isolates to be resistant to clarithromycin, levofloxacin, and metronidazole, respectively. Moreover, they demonstrated significant associations between outpatient levofloxacin and long-acting macrolide use and resistance to levofloxacin (*p* = 0.0013) and clarithromycin (*p* = 0.036), respectively. An updated analysis of 1211 patients in the same settings showed increased rates of *H. pylori* resistance to clarithromycin (21.4%), levofloxacin (15.8%), and metronidazole (38.9%). The authors again found significant associations between community quinolone consumption and levofloxacin resistance (*p* = 0.0002) as well as between macrolide use and clarithromycin resistance (*p* = 0.0003) [37].

Clinical cohort studies eliciting antibiotic use history via medical record review or survey have shown that patients with prior use of metronidazole, macrolides, or fluoroquinolones have increased rates of culture-based resistance to each of these agents and suffer higher rates of *H. pylori* treatment failure [26,33]. Data summarizing the observed relationships between prior antibiotic exposure and rates of *H. pylori* antibiotic resistance are shown in Table 1. Among 125 *H. pylori* isolates from Alaskan Native patients, 30% were resistant to clarithromycin and 66% were resistant to metronidazole. The risk of clarithromycin resistance increased with each prior course of macrolides, from 7% among patients with no prior macrolide use to 50% among patients with three to four prior courses and 80% among patients with at least five prior courses, respectively (*p* < 0.001). Patients with metronidazole-resistant isolates had significantly higher rates of prior metronidazole prescription (60%) compared to those with susceptible isolates (10%, *p* < 0.001) [26]. In a study of 128 isolates from male U.S. veterans by Shiota et al. [33], 31.3% were resistant to levofloxacin, 20.3% were resistant to metronidazole, and 16.4% were resistant to clarithromycin. Clarithromycin resistance was more than twice as frequent among patients reporting prior macrolide use compared to those reporting no prior use (30.0% vs. 12.2%, *p* = 0.02). Multivariate analysis with adjustment for age, race, and year of sample isolation showed that prior use of macrolides was associated with greater odds of resistance to any antibiotic (OR = 3.92, 95% CI 1.39–11.07). Prior fluoroquinolone use was also associated with increased resistance to levofloxacin (43.6% vs. 25.8%, *p* = 0.046 and OR = 3.49, 95% CI 1.30–9.36) [33].

**Table 1:** Antibiotic-resistant *H. pylori* by prior antibiotic exposure.

	Patients with Resistance to:			
Study	CLR	MTZ	FQ ^a^	Any
Shiota et al. [33]				
Prior macrolide use (*n* = 30)	30.0%	26.7%	36.7%	60.0%
No macrolide use (*n* = 98)	12.2%	18.4%	29.6%	45.9%
*p*-value	0.02	0.32	0.46	0.18
Odds ratio ^b^	-	-	-	3.92
95% CI	-	-	-	(1.39–11.07)
Prior fluoroquinolone use (*n* = 39)	23.1%	30.8%	43.6%	64.1%
No fluoroquinolone use (*n* = 89)	13.5%	15.7%	25.8%	42.7%
*p*-value	0.18	0.05	0.046	0.03
Odds ratio ^b^	-	-	3.49	-
95% CI	-	-	(1.30–9.36)	-
Prior treatment of *H. pylori* (*n* = 7)	71.4%	42.9%	71.4%	100.0%
No prior treatment (*n* = 110)	14.5%	17.3%	29.1%	45.5%
*p*-value	0.002	0.12	0.03	0.01
Odds ratio ^b^	11.37	4.64	3.83	-
95% CI	(1.79–72.21)	(0.86–24.92)	(0.60–24.36)	-
McMahon et al. [26]				
No prior macrolide use (*n* = 41)	7%	-	-	-
1 course of macrolides (*n* = 29)	28%	-	-	-
2 courses of macrolides (*n* = 22)	23%	-	-	-
3–4 courses of macrolides (*n* = 18)	50%	-	-	-
5 courses of macrolides (*n* = 15)	80%	-	-	-
*p*-value	<0.001	-	-	-
McNulty et al. [27]				
No prior clarithromycin use (*n* = 103)	7%	-	-	-
1 course of clarithromycin (*n* = 21)	19%	-	-	-
2+ courses of clarithromycin (*n* = 8)	25%	-	-	-
*p*-value	0.12	-	-	-
Relative risk ^c^	1.5	-	-	-
95% CI	(0.92–2.41)	-	-	-
No prior metronidazole use (*n* = 114)	-	28%	-	-
1 course of metronidazole (*n* = 13)	-	38%	-	-
2+ courses of metronidazole (*n* = 5)	-	100%	-	-
*p*-value	-	0.002	-	-
Relative risk ^c^	-	1.6	-	-
95% CI	-	(1.46–1.75)	-	-
No prior quinolone use (*n* = 114)	-	-	4%	-
1 course of quinolones (*n* = 7)	-	-	14%	-
2+ courses of quinolones (*n* = 11)	-	-	27%	-
*p*-value	-	-	0.01	-
Relative risk ^c^	-	-	1.8	-
95% CI	-	-	(1.24–2.49)	-
Bai et al. [38]				
Prior treatment of *H. pylori* (*n* = 37)	51.4%	83.8%	78.4%	-
No prior treatment (*n* = 144)	25.7%	55.6%	72.9%	-
*p*-value	0.005	0.002	0.674	-
Odds ratio ^d^	3.354	3.836	NS	-
95% CI	(1.514–7.429)	(1.456–10.109)	NS	-

^a^ Tested fluoroquinolone was moxifloxacin in Bai et al., and levofloxacin in all other studies. ^b^ Odds ratio for prior antibiotic:no antibiotic are reported from multivariate analysis with adjustment for age, race, and year of isolation. ^c^ Expressed as the ratio of the risk of being resistant per unit increase in number of courses. ^d^ Odds ratio for prior treatment:no treatment are reported from multivariate analysis with adjustment for age and gender. CLR = clarithromycin; MTZ = metronidazole; FQ = fluoroquinolone; Any = clarithromycin, metronidazole, levofloxacin, or tetracycline.

In a study of 2063 *H. pylori* isolates from patients at three sites in the United Kingdom, rates of resistance to clarithromycin, levofloxacin, and metronidazole varied widely, at 3–68%, 1–17%, and 22–88%, respectively. Each prior course of quinolone increased the risk of levofloxacin resistance from 4% among quinolone-naïve patients to 27% among patients with at least two prior courses of quinolones (RR = 1.8, 95% CI 1.24–2.49). Risk of metronidazole resistance similarly increased with each prior course of metronidazole, from 28% among metronidazole-naïve patients to 100% among patients with at least two prior courses (RR = 1.6, 95% CI 1.46–1.75). Although the risk of clarithromycin resistance increased numerically with each prior course of clarithromycin, from 7% among clarithromycin-naïve patients to 25% among those with at least two prior courses, this did not reach statistical significance (RR = 1.5, 95% CI 0.92–2.41). Similarly, although there was a trend towards more frequent tetracycline resistance among patients with one prior tetracycline or doxycycline course compared to naïve patients (20% vs. 0.9%) none of the patients with at least two prior tetracycline courses had tetracycline-resistant isolates, and there was no significant association (RR = 1.6, 95% CI 0.74–3.50). Rates of amoxicillin resistance were comparable between amoxicillin-naïve patients (0%) and those with at least two prior courses (2%), with no significant association (RR = 1.0, 95% CI 0.48–2.04) [27].

The increased incidences of antibiotic resistance among patients with prior antibiotic exposure are reflected in higher rates of eradication failure among these patients. Among 212 patients with *H. pylori* infection treated with clarithromycin-based regimens in Spain, eradication rates were significantly lower among patients with previous macrolide use compared to patients without (60.8% vs. 92.9%, *p* < 0.0001 and 85.7% vs. 98.2% *p* = 0.024 for standard triple and concomitant therapies, respectively) [39]. A larger study of 7842 patients in Israel found significantly lower odds of eradication success with clarithromycin-based standard triple therapy among patients with prior macrolide exposure (OR = 0.62, 95% CI 0.55–0.70), although there was no effect on the success of concomitant or sequential therapies. There appeared to be slight dose-dependent effects, as prior exposure to higher cumulative doses of clarithromycin and roxithromycin were associated with marginally but statistically significantly lower odds of eradication success (ORs = 0.99988, 95% CI 0.99982–0.99996 and OR = 0.99981, 95% CI 0.99971–0.99992, respectively) [40]. A retrospective analysis of 3181 patients in South Korea showed significantly lower eradication rates with the standard clarithromycin-based triple therapy among patients with prior clarithromycin or macrolide use (64.1% vs. 85.9%, *p* < 0.001 and 71.2% vs. 85.7%, *p* < 0.001, respectively). Multivariate analysis demonstrated significantly greater odds of eradication failure with prior clarithromycin or other macrolide use (OR = 4.445, 95% CI 2.619–7.544 and OR = 2.407, 95% CI 1.476–3.928, respectively). The risk of clarithromycin-based treatment failure was substantially increased (44.8% vs. 29.3%, *p* = 0.047) in patients with a history of macrolide use for more than 2 weeks compared to those exposed for <2 weeks, corroborating the dose-response effect seen in other studies [28]. The relationship between timing of prior antibiotic exposure and *H. pylori* treatment failure is not known, although a recent retrospective analysis of 120,787 patients in China found that odds of clarithromycin-based regimen failure were consistently and stably higher among patients with prior exposure to macrolides within 180 days (OR = 1.64, 95% CI 1.43–1.87), 1 year (OR = 1.89, 95% CI 1.70–2.10), and 3 years (OR = 2.03, 95% CI 1.88–2.19). Interestingly, antibiotic exposure within the 30 days prior to *H. pylori* treatment was not associated with a higher rate of eradication failure, raising the possibility of a defined window period which may be required for resistance to develop [41].

Unsurprisingly, antibiotic-resistant isolates tend to be more common among patients with prior *H. pylori* treatment, reflecting failure of these agents. Shiota et al. [33] found increased odds of clarithromycin resistance (OR = 11.37, 95% CI 1.79–72.21) among patients with prior *H. pylori* treatment. Univariate analysis showed significantly more previously treated patients with levofloxacin resistance compared to treatment-naïve patients (71.4% vs. 29.1%, *p* = 0.03), but this difference did not remain significant in multivariate analysis (OR = 3.83, 95% CI 0.60–24.36). Metronidazole resistance was numerically but not significantly higher among previously treated patients (42.9% vs. 17.3%, *p* = 0.12 and OR = 4.64, 95% CI 0.86–24.92). Bai et al. [38] similarly observed more clarithromycin resistance (51.4% vs. 25.7%, *p* = 0.005 and OR = 3.354, 95% CI 1.514–7.429) among previously treated patients, as well as significantly more metronidazole resistance (83.8% vs. 55.6%, *p* = 0.002 and OR = 3.836, 95% CI 1.456–10.109) but no difference in moxifloxacin resistance.

Intuitively, prior treatment of *H. pylori* with the same agent used in subsequent regimens may predispose the individual to harbor resistant strains. Cross-resistance, or antibiotic resistance in the setting of prior exposure to a different antibiotic, may also play a role. A study of 65 isolates from patients in the USA with refractory *H. pylori* found that resistance to levofloxacin, metronidazole, or clarithromycin was associated with resistance to either of the other two antibiotics (*p* < 0.05 for all) [42]. Guo et al. [41] also observed greater odds of clarithromycin-based regimen treatment failure among patients with any prior penicillin (OR = 1.26, 95% CI 1.21–1.31), tetracycline (OR = 1.51, 95% CI 1.31–1.74), nitroimidazole (OR = 1.22, 95% CI 1.13–1.32), and quinolone (OR = 1.39, 95% CI 1.30–1.49) use within the prior 3 years. Overall, prior exposure to any antibiotic within 180 days (OR = 1.18, 95% CI 1.13–1.24), 1 year (OR = 1.26, 95% CI 1.20–1.31), and 3 years (OR = 1.30, 95% CI 1.25–1.35) was associated with greater odds of regimen failure.

The mechanism by which prior antibiotic exposure contributes to antibiotic-resistant *H. pylori* is intuitive – resistance develops when strains mutate in the face of a strong selection pressure, such as an antibiotic. Hypermutable strains are common among *H. pylori*, and this predisposition to high mutation frequencies may account for the organism’s rapid development of resistance following antibiotic therapy [43]. Given the high rates of subsequent antibiotic resistance, guidelines recommend that patients who fail *H. pylori* regimens containing clarithromycin, fluoroquinolones or metronidazole should not repeat therapy with these agents [18]. Currently, rates of resistance to amoxicillin, tetracycline, and bismuth are low, and as there are no convincing data to suggest that prior exposure to these agents increases the risk of resistance, guidelines permit repeat therapy with these agents [4,18].

### 2.2. Age

Several studies have evaluated rates of antibiotic-resistant *H. pylori* by age (Table 2).

**Table 2:** Antibiotic-resistant *H. pylori* by age group.

	Patients with Resistance to:			
Study	CLR	MTZ	FQ ^a^	Any
Zullo et al. [29]				
>45 years (*n* = 142)	19.0%	28.2%	28.4%	-
<45 years (*n* = 113)	14.1%	28.3%	14.4%	-
*p*-value	NS	NS	0.048	-
Megraud et al. 2013 [30]				
Age < 50 years (*n* = 847)	-	-	-	-
Age > 50 years (*n* = 1046)	-	-	-	-
*p*-value	0.305	0.329	0.012	-
Odds ratio ^b^	1.13	0.91	1.51	-
95% CI	(0.89–1.44)	(0.75–1.10)	(1.09–2.09)	-
Shiota et al. [33]				
Age < 60 years (*n* = 51)	11.8%	21.6%	23.5%	51.0%
Age ≥ 60 years (*n* = 77)	19.5%	19.5%	36.4%	48.1%
*p*-value	0.25	0.77	0.13	0.75
Odds ratio ^c^	2.16	0.74	2.34	1.04
95% CI	(0.67–6.99)	(0.26–2.10)	(0.87–6.28)	(0.44–2.48)
Megraud et al. 2021 [37]				
Age < 50 years (*n* = NR)	-	-	-	
Age ≥ 50 years (*n* = NR)	-	-	-	
*p*-value	0.538	0.841	0.608	-
Odds ratio ^d^	0.87	1.04	0.89	-
95% CI	(0.56–1.36)	(0.70–1.54)	(0.57–1.39)	-
Bai et al. [38]				
≤35 years (*n* = 55)	34.5	56.4	72.7	-
35–60 years (*n* = 98)	30.6	63.3	73.5	-
≥60 years (*n* = 28)	25.0	64.3	78.6	-
*p*-value	0.670	0.660	0.833	-
Ji et al. [44]				
Age < 20 years (*n* = 114)	16.67%	-	6.14%	-
Age 21–30 (*n* = 705)	14.61%	-	9.65%	-
Age 31–40 (*n* = 1294)	19.71%	-	19.78%	-
Age 41–50 (*n* = 2330)	20.94%	-	22.19%	-
Age 51–60 (*n* = 2977)	15.38%	-	18.41%	-
Age 61–70 (*n* = 1976)	16.75%	-	21.31%	-
Age 71–80 (*n* = 291)	23.02%	-	29.9%	-

^a^ Tested fluoroquinolone was moxifloxacin in Bai et al., and levofloxacin in all other studies. Fluoroquinolone resistance data were reported for 246 out of 255 patients by Zullo et al. ^b^ Odds ratios for >50:<50 years are reported from univariate analyses of clarithromycin and metronidazole and from multivariate analysis of levofloxacin. ^c^ Odds ratio for ≥60:<60 years are reported from multivariate analysis with adjustment for age, race, and year of isolation. ^d^ Odds ratios for >50:<50 years are reported from multivariate analysis. CLR = clarithromycin; MTZ = metronidazole; FQ = fluoroquinolone; NR = not reported.

A study of 255 *H. pylori* patients in Italy by Zullo et al. [29] found levofloxacin resistance nearly twice as frequently among patients 45 years or older compared to those younger than 45 (28.4% vs. 14.4%, *p* = 0.048), which the authors speculated may be due to higher use of quinolones for urinary tract infections among older patients. They did not observe differences with respect to age for resistance to clarithromycin or metronidazole (19% vs. 14.1% and 28.2% vs. 28.3%, respectively). Megraud et al. [30] similarly found increased odds of levofloxacin resistance among European patients of 50 years and older compared to those younger than 50, however an updated analysis found no difference in rates of resistance between the two groups [37]. A study of 9687 isolates from patients in China also found increased levofloxacin resistance with age, reaching a peak of 29.9% among patients aged 71–80. Patients age 30 or younger had significantly lower rates of resistance (6.14–9.65%) compared to those in older age groups (18.41–29.9%; *p* < 0.001). The authors again attributed this association to a higher incidence of urinary tract infections among older adults. While clarithromycin resistance also peaked among patients aged 71–80 at 23.02%, a similar peak was observed among patients aged 31–50 (19.71–20.94%), and it was speculated that these associations may be attributed to historical patterns of macrolide use among these groups [44]. Studies by Bai et al. [38] and Shiota et al. [33] showed similar trends in increasing rates of fluoroquinolone resistance among those 60 years or older (78.6% vs. 73.2% and 36.4% vs. 23.5%, respectively), although these differences did not reach statistical significance.

A meta-analysis comprising 66,142 isolates from 65 countries by Savoldi et al. [4] demonstrated that in most World Health Organization-defined regions, children have lower rates of antibiotic resistant *H. pylori* compared to adults. Surprisingly, isolates from children in the Americas, Eastern Mediterranean, and Western Pacific regions were more likely resistant to (i) metronidazole (40% vs. 22%), (ii) metronidazole (81% vs. 61%) and levofloxacin (29% vs. 18%), and (iii) clarithromycin (85% vs. 32%), respectively, than those from adults (*p* < 0.05 for all). These increased rates of antibiotic-resistant *H. pylori* among children from each of these regions were noted to be based on a small number of studies, although it was hypothesized that the increased metronidazole resistance in the Americas may be related to the high prevalence of over-the-counter antibiotic use among children and adolescents in Brazil.

The data regarding *H. pylori* eradication failure rates as a function of age are conflicting. Lim et al. [28] found that eradication failure rates with clarithromycin-based triple therapy were significantly higher among patients older than 60 years (18.3% vs. 13.9%, *p* = 0.007). This association persisted in the multivariate logistic regression model (OR 1.326, 95% CI 1.046–1.681). On the contrary, in their multivariate analysis Boltin et al. [40] found that younger age was associated with failure of clarithromycin-based regimens independently of macrolide exposure. Broutet et al. [34] similarly found age over 60 to be protective against eradication failure in their adjusted analysis (OR = 0.6, 95% CI 0.5–0.8). The authors hypothesized that this difference may be attributable to characteristics independent of antibiotic resistance encountered more frequently in older patients, namely, the histologic features of intestinal metaplasia and atrophic chronic gastritis, which provide an inhospitable environment for the bacteria and promote acid suppression, respectively. Data regarding the impact of age on eradication success with quinolone-containing regimens are more limited. In a South Korean study of 98 patients who failed non-bismuth quadruple therapy, the authors observed no significant difference in eradication success with a moxifloxacin-based regimen between those >60 and ≤ 60 years old (68.4% vs. 61.7%) [45]. More study is needed to clarify the impact of quinolone, and particularly levofloxacin-resistant *H. pylori* on eradication success among older patients.

While cumulative antibiotic use—in particular fluoroquinolones—may alter the prevalence of antibiotic-resistant *H. pylori*, this relationship may also be mediated by mechanisms that are not yet fully elucidated. We observed that *H. pylori* infection is associated with significantly decreased fecal microbial diversity relative to controls over the age of 40 years [46]. Our results suggest that *H. pylori* may cause an alteration of the fecal microbiome that progresses with age. The decreased diversity of gastric gut microbiota may be due to the ability of *H. pylori* to outcompete other species, secretion of antibacterial peptides, and local pH alterations; however, the exact mechanism is not clear, particularly in the distal gut [47,48,49,50]. Age-related decrease in gut microbiome diversity may be due to the influence of diet or chronic inflammation. These patients may be more prone to *H. pylori* infection, particularly by mid-adulthood. In our previous study of 2014 *H. pylori* patients, we observed that 80% of our patients were above the age of 40 years, consistent with studies showing increased prevalence of *H. pylori* infection with age in both developing and developed countries [24,51]. Yang et al. [52] characterized the altered composition of microbiome induced by (i) *H. pylori* infection and (ii) gastritis and suggested that the changes in gut microbiome caused by *H. pylori* may be related to drug resistance. Although it warrants further study, there is a strong possibility that the impact of *H. pylori* on the surrounding gut microbiome may contribute to the proliferation of resistant strains.

Overall, the data suggest increased risk of levofloxacin resistance among older adults with *H. pylori*. Clarithromycin and metronidazole resistance do not appear to be consistently associated with age; however, there may be age-related differences that reflect regional patterns of antibiotic use, as described above [4]. Most guidelines recommend avoidance of levofloxacin-based regimens as first-line therapy due to concerns about increasing rates of resistance [17]. These concerns are particularly relevant in older adults given the demonstrably greater rates of resistance. Local *H. pylori* eradication surveillance efforts should pay close attention to rates of resistance among older patients, and clinicians may find levofloxacin susceptibility testing particularly useful when considering prescription of this agent for second or third-line therapy in older patients.

### 2.3. Gender/Sex

Antibiotic-resistant *H. pylori* may be more common in women. Table 3 shows rates of antibiotic-resistant *H. pylori* and odds ratios among women compared to men from studies reporting data by gender or sex. Note that while these terms are increasingly recognized to not be interchangeable, there was significant heterogeneity in the language used by authors of our identified studies, with some using the term “gender”, “sex”, “male”, “female”, “men”, “women”, or some combination of terms. We use both “gender” and “sex” to capture the heterogeneity in reporting of this variable in the reviewed studies.

**Table 3:** Antibiotic-resistant *H. pylori* by gender or sex.

	Patients with Resistance to:		
Study	CLR	MTZ	FQ ^a^
Zullo et al. [29]			
Male (*n* = 94)	17.0%	24.4%	20.1%
Female (*n* = 161)	16.7%	30.4%	16.7%
*p*-value	NS	NS	NS
Megraud et al. 2013 [30]			
Male (*n* = 1077)	-	-	-
Female (*n* = 1127)	-	-	-
*p*-value	0.006	0.001	0.59
Odds ratio ^b^	1.40	1.63	1.07
95% CI	(1.10–1.78)	(1.28–2.09)	(0.82–1.39)
Tveit et al. [31]			
Male (*n* = 271)	24%	32%	16%
Female (*n* = 260)	37%	52%	24%
*p*-value	<0.05	<0.05	NS
Megraud et al. 2021 [37]			
Men (*n* = NR)	-	-	-
Women (*n* = NR)	-	-	-
*p*-value	0.604	0.596	0.526
Odds ratio ^c^	0.89	1.11	0.87
95% CI	(0.57–1.38)	(0.76–1.63)	(0.56–1.35)
Bai et al. [38]			
Male (*n* = 108)	29.6%	61.1%	76.9%
Female (*n* = 73)	32.9%	61.6%	69.9%
*p*-value	0.743	1.000	0.305
Shao et al. [53] ^d^			
Male (*n* = 1225)	20.08%	89.97%	21.80%
Female (*n* = 1058)	25.80%	95.01%	28.17%
*p*-value	0.001	0.012	<0.001

^a^ Tested fluoroquinolone was moxifloxacin in Bai et al., and levofloxacin in all other studies. Fluoroquinolone resistance data were reported for 246 out of 255 patients by Zullo et al. Levofloxacin resistance data were reported for 155 out of 531 patients by Tveit et al. ^b^ Odds ratios for female:male are reported from univariate analyses of clarithromycin and levofloxacin and from multivariate analysis of metronidazole. ^c^ Odds ratios for women:men are reported from multivariate analysis. ^d^ Metronidazole resistance data were reported for 750 out of 2283 patients by Shao et al. CLR = clarithromycin; MTZ = metronidazole; LVX = levofloxacin. NR = not reported.

Significantly higher rates of metronidazole, clarithromycin, and quinolone resistance have been observed in women in cohort studies. McMahon et al. [26] found that Alaskan Natives harboring metronidazole-resistant isolates were more likely to be women (76% vs. 45%, *p* < 0.001). The authors attributed this difference to higher metronidazole use among women, because after controlling for metronidazole use, there was no association between sex and resistance via logistic regression (*p* = 0.21). In a similar population, Tveit et al. [31] found significantly higher rates of *H. pylori* resistance to metronidazole (52% vs. 32%, *p* < 0.05, OR = 2.6) and clarithromycin (37% vs. 24%, *p* < 0.05, OR = 1.7) compared to men. Dual resistance to both clarithromycin and metronidazole was also more common among women (21% vs. 10%, *p* = 0.0004, OR = 2.4). The authors again cited increased rates of metronidazole use among women as a possible mechanism for these differences. Rates of antibiotic resistance among the overall Alaskan Native population were much higher than those reported for other US populations, which may limit the generalizability of these associations with gender. No significant differences were observed in rates of levofloxacin or amoxicillin resistance between men and women. In an Italian study involving 248 with *H. pylori*, female sex was identified as a predictor of antibiotic resistance, primarily to metronidazole (OR = 2.74, 95% CI 1.03–7.27), supporting the notion that this relationship may not be confined to specific populations [54].

Zullo et al. [29] showed similarly increased rates of metronidazole resistance among women compared to men (30.4% vs. 24.4%), although this difference did not reach statistical significance. In their 2013 analysis, Megraud et al. [30] also observed increased odds of metronidazole resistance among European women via multivariate analysis (OR = 1.63, 95% CI 1.28–2.09), while a univariate analysis showed increased odds of clarithromycin resistance (OR = 1.40, 95% CI 1.10–1.78) [30]. In the group’s more recent analysis, however, odds of resistance to clarithromycin, metronidazole, and levofloxacin were not significantly different by gender [37].

In an analysis of 2283 samples in China, higher rates of resistance to clarithromycin (25.80% vs. 20.08%, *p* = 0.001), levofloxacin (28.17% vs. 21.80%, *p* < 0.001), cefetamet (98.43% vs. 95.53%, *p* = 0.046), azithromycin (88.98% vs. 82.11%, *p* = 0.009), metronidazole (95.01% vs. 89.97%, *p* = 0.012), and ciprofloxacin (49.61% vs. 36.59%, *p* < 0.001) were observed among isolates from women. The authors hypothesized that these differences may be related to an increased susceptibility to infections and greater willingness to receive treatment among women, as well as hormonal and genetic differences. This study was the only one we identified that found significantly increased rates of quinolone resistance among women. Whether this phenomenon is unique to the studied population and the mechanisms to account for this are unclear. Higher utilization of quinolones for urinary tract infections among women could conceivably be a culprit. Rifampicin (2.36% vs. 3.31%) and furazolidone (1.51% vs. 1.47%), which are being increasingly employed for *H. pylori* treatment regimens in certain parts of the world, did not demonstrate significant differences in rates of resistance between gender groups [53].

A recent study of 5249 patients in Japan found a roughly 10% higher rate of clarithromycin resistance among women (*p* < 0.0001) but found no difference in the rate of metronidazole resistance, although overall metronidazole resistance in the population was very low (2.31%). The authors used a higher breakpoint to define metronidazole resistance at MIC ≥ 16 μg/mL compared to other studies which used MIC ≥ 8 μg/mL as the threshold, but they reported that there were no potentially ambiguous cases with MICs between 8.1 and 15.9 μg/mL [55]. Interestingly, both Zullo et al. [29] and Bai et al. [38] found trends towards lower rates of fluoroquinolone resistance among women (16.7% vs. 20.1% and 69.9% vs. 76.9%, respectively), but these did not reach statistical significance.

The relationship between increased rates of antibiotic-resistant *H. pylori* and eradication success among women is less clear, in part because of inconsistent data regarding the impact of metronidazole resistance on treatment failure. Recent data do suggest, however, that metronidazole resistance is indeed associated with eradication failure, particularly when coincident with clarithromycin resistance (OR = 31.432, 95% CI 3.094–319.266) [56]. There are also data supporting higher rates of treatment failure among women. Kumar et al. [42] found that 74% of patients referred for refractory *H. pylori* at their US tertiary care center were women. Lim et al. [28] observed higher rates of eradication failure with clarithromycin-based triple therapy among women (17.9% vs. 12.7%, *p* < 0.001), and this association persisted in their adjusted multivariate analysis (OR = 1.339, 95% CI 1.094–1.639). Interestingly, Broutet et al. [34] did not identify significant differences in treatment success as a function of gender in their analysis of French patients. It is unclear whether regional patterns of clarithromycin and/or metronidazole use could account for this finding in contrast with other studies.

Overall, the data suggest higher rates of clarithromycin and metronidazole resistance among women. The mechanisms for these associations are unclear but may reflect greater use of antibiotics, including metronidazole for gynecologic and quinolones for urinary tract infections. Clinicians should be alert to increased risk of clarithromycin and metronidazole resistance among women with *H. pylori* when choosing empiric therapy and consider susceptibility testing to these agents when available. Local surveillance data will be useful in determining how these associations vary by population and in resolving conflicting data regarding rates of fluoroquinolone resistance and the impact of metronidazole resistance on eradication success among women.

### 2.4. Race/Ethnicity

*H. pylori* infection is more common among certain racial and ethnic groups even within a particular geographic region [57,58]. Rates of *H. pylori* antibiotic resistance may also vary by racial and ethnic group, but the limited available data regarding the association of race with antibiotic resistance do not paint a consistent picture. A multicenter U.S. study of isolates from 347 patients by Duck et al. [32] suggested that black patients were more likely to harbor *H. pylori* with resistance to at least one antibiotic, including amoxicillin, tetracycline, clarithromycin, or metronidazole (HR = 2.1, 95% CI 1.1–3.8). This association was evident even in the multivariate model with conditioning on geographic location, previous *H. pylori* treatment, and antacid use. This relationship was thought to reflect persistent transmission of resistant strains within the studied black communities, although it was unclear to what extent repeated in vivo induction of resistance also played a role [32].

The study by Shiota et al. [33] of US male veterans reported trends towards higher rates of resistance to clarithromycin (26.1% vs. 17.9% and OR = 1.47, 95% CI 0.35–6.11) and levofloxacin (43.5% vs. 28.2% and OR = 2.48, 95% CI 0.65–9.52) among Hispanic patients compared to white patients, but these differences did not reach statistical significance. The authors cited higher rates of inappropriate antibiotic use and low health literacy described by Cespedes et al. [59] as possible mechanisms accounting for these trends among Hispanic patients in the US [33].

Racial and ethnic differences in rates of antibiotic resistant *H. pylori* could reflect disparities in access to health care, locoregional patterns of antibiotic use, and broader institutional determinants of disease transmission, but genetic and cultural influences may have adjunctive roles. More research is needed to better define these differences and elucidate the underlying factors at play. Notably, we did not identify data on the impact of race or ethnicity on eradication rates and cannot draw conclusions in this regard. Nevertheless, clinicians should be aware of potential differences when judging the likelihood of antibiotic resistance and choosing appropriate *H. pylori* therapy. Susceptibility testing may be particularly helpful for black and Hispanic patients, especially when considering the use of levofloxacin-based regimens as second or third-line therapy. Local surveillance efforts tracking *H. pylori* antibiotic resistance by race will be crucial to evaluate the impact on eradication rates and help address health disparities given the high burden of this infection among racial minorities and those facing socioeconomic barriers.

### 2.5. Alcohol Consumption

Data from various studies suggest that less alcohol consumption is associated with increased rates of metronidazole-resistant *H. pylori* and vice versa. It was observed that patients who reported current or previous alcohol consumption had significantly lower rates of resistance to metronidazole (54.5% among never drinkers compared to 12.8% among former drinkers and 18.0% among current drinkers, respectively; *p* = 0.01). This association remained in multivariate analysis adjusted for age, race, and year of sample isolation (OR = 0.12, 95% CI 0.02–0.65 and OR = 0.19, 95% CI 0.04–0.94 for former and current drinkers compared to never drank, respectively). The authors suggested that clinicians may be less inclined to prescribe metronidazole in patients who consume alcohol due to the disulfiram-like reaction that can result from their co-ingestion, and metronidazole resistance would thus be less likely to develop in this population. Levofloxacin resistance was numerically higher among patients reporting current or previous alcohol use (9.1% among never drinkers compared to 31.9% among former drinkers and 34.4% among current drinkers), but these differences did not reach statistical significance [33]. Pilotto et al. [54] also reported that less alcohol consumption was associated with antibiotic resistance via univariate analysis. However, under multivariate analysis controlling for additional risk factors, alcohol consumption was not significantly associated with antibiotic resistance, suggesting that it may simply be a proxy for other factors associated with resistance, rather than an independent determinant [54].

While patients who consume alcohol may be less likely to harbor antibiotic resistant *H. pylori*, alcohol consumption is unlikely to improve eradication rates. In fact, data from a study of 992 Chinese patients treated with furazolidone and amoxicillin-based quadruple therapy suggest that alcohol consumption is associated with increased rates of treatment failure (OR = 4.4, 95% CI 1.5–12.3). The authors hypothesized that this could be due to adverse reactions that have a negative impact on adherence [60].

Patients with no history of alcohol use may be good candidates for metronidazole resistance testing, especially when choosing second- or third-line therapies. Local surveillance efforts should aim to further define the impact of individual alcohol use on eradication success rates with metronidazole-containing regimens. Standard triple therapy, high-dose amoxicillin dual therapy, or levofloxacin-based regimens may prove more effective choices in patients without prior alcohol use.

### 2.6. Non-Ulcer Dyspepsia

Data from multiple studies suggest that patients with non-ulcer dyspepsia have higher rates of antibiotic-resistant *H. pylori* compared to patients with peptic ulcers. Table 4 shows rates of antibiotic resistance grouped by the presence or absence of a peptic ulcer. Data have consistently shown significantly higher rates of clarithromycin resistance among patients with non-ulcer dyspepsia compared to those with ulcers across many different parts of the world. Among 402 *H. pylori* isolates from Chinese patients, clarithromycin resistance was significantly more common among patients with non-ulcer dyspepsia compared to those with duodenal ulcers (13.6% vs. 6.4%, *p* = 0.015). Such differences were not seen for amoxicillin and metronidazole resistance [61]. Broutet et al. [34] compared isolates from 257 patients with non-ulcer dyspepsia to 179 patients with ulcers in France. The former group had a three-fold higher incidence of clarithromycin resistance (16.7% vs. 5.6%, *p* = 0.0005).

In Italy and China, Zullo et al. [29] and Bai et al. [38] similarly found significantly higher rates of clarithromycin resistance among patients with chronic gastritis or NUD compared to those with peptic ulcers (19.1% vs. 0% and 36.8 vs. 20.9%, respectively, *p* = 0.02 for both). However, this association did not hold in the latter study after adjustment for age and gender (OR = 2.101, 95% CI 0.969–4.169). In their large European analysis, Megraud et al. [30] found the odds of clarithromycin resistance among patients with peptic ulcers to be half of those without (OR = 0.5 95% CI 0.32–0.77). While data suggest that clarithromycin resistance is higher among patients with non-ulcer dyspepsia, there is less evidence of differences in rates of resistance to other antibiotics. Megraud et al. [30] did find lower odds of levofloxacin resistance among patients with peptic ulcers (OR = 0.65, 95% CI 0.42–0.99), but this difference has not been observed in other studies.

Higher rates of clarithromycin-resistant *H. pylori* among patients with non-ulcer dyspepsia are thought to be related to the higher rates of cagA-positive strains among patients with peptic ulcer disease [62]. The cagA gene is associated with an increased mucosal inflammatory response, however cagA-positive *H. pylori* strains are associated with higher eradication success rates. Possible mechanisms for greater eradication rates include the shorter generation time of cagA-positive strains, maximizing the efficacy of antibiotics that are most active during cell division, or increased blood flow in the setting of heightened inflammation, facilitating greater antibiotic delivery [63].

Lower prevalence of antibiotic-resistant *H. pylori* among patients with peptic ulcer disease are reflected in consistently higher rates of eradication success among this group. In parallel with their observation of increased clarithromycin resistance among patients with non-ulcer dyspepsia compared to those with duodenal ulcers, Wong et al. [61] also found a significantly higher eradication rate among patients with duodenal ulcers (91.3% vs. 84.3%, *p* = 0.011). In the analysis of 2751 patients in France by Broutet et al. [34], 21.9% of patients with duodenal ulcers suffered eradication failure, compared to 33.7% of patients with non-ulcer dyspepsia (*p* < 10^−6^). In their adjusted multivariable analysis, non-ulcer dyspepsia was a risk factor for eradication failure (OR = 1.2, 95% CI 1.0–1.4), and the authors attributed this association to the higher incidence of clarithromycin resistance observed among patients with non-ulcer dyspepsia. Lim et al. [28] similarly found that patients with a gastroduodenal ulcer were significantly more likely to achieve eradication success with clarithromycin-based triple therapy (88.1% vs. 81.9%, *p* < 0.001). Odds of eradication failure were lower among patients with an ulcer via multivariate analysis (OR = 0.641, 95% CI 0.523–0.784). Authors of both studies cited the higher frequency of cagA and vacAs1 allele-positive *H. pylori* strains in patients with peptic ulcers as a possible mechanism to account for the association between ulcers, lower resistance rates, and greater eradication success. In their Spanish study, Muñoz-Gómez et al. [39] also observed significantly higher rates of eradication success with standard triple and concomitant therapies among patients with peptic ulcers compared to those without (100% vs. 80.6%, *p* = 0.025). Interestingly, Lim et al. [45] found no difference in eradication success rates with moxifloxacin-containing triple therapy among those with peptic ulcers compared to those without. It is not clear whether this departure from results found in other studies reflects a difference inherent in moxifloxacin’s activity in patients with non-ulcer dyspepsia or could be attributed simply to the type of *H. pylori* strains predominant in this population.

Overall, clarithromycin resistance and clarithromycin-based regimen failure are more common among patients without peptic ulcers. This relationship is rather consistent across populations, but the acquisition of local surveillance data should clarify whether clarithromycin-containing regimens are appropriate for empiric use among patients with non-ulcer dyspepsia and if certain regimens serve as better options. Clarithromycin susceptibility testing may be of particular use when considering such therapies for patients with this phenotype. Further study should also evaluate the relationships between peptic ulcer disease and resistance to other agents such as fluoroquinolones, along with the impact on treatment failure.

**Table 4:** Antibiotic-resistant *H. pylori* by peptic ulcer status.

	Patients with Resistance to:		
Study	CLR	MTZ	FQ ^a^
Zullo et al. [29]			
NUD (*n* = 226)	19.1	28.3	18.4
PUD (*n* = 29)	0	27.6	13.8
*p*-value	0.02	NS	NS
Megraud et al. 2013 [30]			
NUD (*n* = 1254)	-	-	-
PUD (*n* = 377)	-	-	-
*p*-value	0.002	0.233	0.046
Odds ratio ^b^	0.5	0.85	0.65
95% CI	(0.32–0.77)	(0.65–1.11)	(0.42–0.99)
Broutet et al. [34]			
NUD (*n* = 257)	16.7	-	-
PUD (*n* = 179)	5.6	-	-
*p*-value	0.0005	-	-
Megraud et al. 2021 [37]			
Normal (*n* = NR)	-	-	-
Ulcer/erosions (*n* = NR)	-	-	-
*p*-value	0.133	0.309	0.103
Odds ratio ^c^	1.75	1.34	1.86
95% CI	(0.84–3.61)	(0.76–2.37)	(0.88–3.94)
Inflammation (*n* = NR)			
*p*-value	0.07	0.921	0.114
Odds ratio **^c^**	1.85	0.97	1.75
95% CI	(0.95–3.61)	(0.58–1.64)	(0.87–3.51)
Bai et al. [38]			
Gastritis (*n* = 114)	36.8	62.3	74.6
Peptic ulcer (*n* = 67)	20.9	59.7	73.1
*p*-value	0.021	0.638	0.727
Odds ratio ^d^	2.101	-	-
95% CI	(0.969–4.169)	-	-
Wong et al. [61]			
NUD (*n* = 198)	13.6%	38.4%	-
Duodenal ulcer (*n* = 204)	6.4%	37.3%	-
*p*-value	0.015	0.815	-

^a^ Tested fluoroquinolone was moxifloxacin in Bai et al., and levofloxacin in all other studies. Fluoroquinolone resistance data were reported for 246 out of 255 patients by Zullo et al. ^b^ Odds ratios for PUD:NUD are reported from multivariate analyses of clarithromycin and levofloxacin and from univariate analysis of metronidazole. ^c^ Odds ratios for ulcer/erosions:normal and inflammation:normal are reported from univariate analysis. ^d^ Odds ratio for chronic gastritis:peptic ulcer are reported from multivariate analysis with adjustment for age and gender. CLR = clarithromycin; MTZ = metronidazole; FQ = fluoroquinolone; NUD = non-ulcer dyspepsia; PUD = peptic ulcer disease. NR = not reported.

### 2.7. Proton Pump Inhibitors (PPIs), Gene Polymorphism, and H. pylori Eradication

Although PPIs have been a cornerstone of *H. pylori* therapy for decades, the mechanisms by which they contribute to *H. pylori* eradication remain poorly understood. It was suggested that PPIs have in vitro, albeit weak, antibacterial effect against *H. pylori* and also may have an indirect effect as it inhibits the gastric acid secretion leading to increased concentrations of antibiotics being used [64]. It was also proposed that due to the structural similarity between proton pumps and efflux pumps, concurrent PPI use can also inhibit the *H. pylori* drug efflux pump system of *H. pylori*, which in turn increases its sensitivity to antibiotics [65]. In their meta-analysis, Suzuki et al. [66] implicated altered metabolism of PPIs as one possible mechanism for lower *H. pylori* eradication rates among smokers, along with other smoking-induced physiological changes such as decreased gastric mucosal blood flow and stimulation of gastric acid secretion. Synergy between PPIs and antibiotics may also be attributed to improved antibiotic stability in the setting of increased gastric pH [67]. Gene polymorphism was also suggested to influence the effectiveness of certain PPIs on *H. pylori* eradication. The pathways for metabolism of PPIs depend on the liver CYP enzymes. Therefore, polymorphisms in the cytochrome P450 CYP2C19 gene can affect the efficacy of PPIs, as these are mainly metabolized through a CYP2C19 channel. Patients who have homozygous wild type (*wt*/*wt*) are said to be the strong metabolic type and clear PPI with higher rates as compared to patients with homozygous mutant allele (mt/mt). The later are poor metabolizers. Thus, patients with the wild-type allele of CYP2C19 are less likely to be able to eradicate *H. pylori*. Zhao et al. [68] showed that the eradication rates are higher in poor metabolizers than in strong metabolizers for PPIs such as omeprazole or lansoprazole that are CYP2C19 metabolic pathway-dependent PPIs. Schwab et al. [69] found similar results in white populations.

Although concurrent PPI use with antibiotics is a mainstay of *H. pylori* therapy, the impact of prior PPI use on eradication rates is the subject of some controversy. Some data suggest that there is a reduction in the eradication rate if PPIs are used prior to antibiotic therapy to treat *H. pylori* infection [70]. It was suggested that this may be because prolonged use of PPI leads to *H. pylori* metastases from the gastric antrum to the body, transforming it into the coccoid form, which is less sensitive to antibiotics [71]. However, another study showed that long term PPI pretreatment did not affect *H. pylori* eradication rate [72]. Moreover, we observed that previous use of PPI actually increased the chance of eradication of *H. pylori* in our patient population [24].

Overall, the relationship between antibiotic resistant *H. pylori* and PPIs remains to be fully elucidated. Given emerging concerns about PPI over-prescription and adverse effects of long-term use, including decreased distal gut microbial diversity observed in our recent analysis of *H. pylori* patients taking PPI prior to eradication therapy, this topic warrants further study [46,73].

## 3. Conclusions

Antibiotic resistance is one of multiple factors implicated in *H. pylori* treatment failure, along with comorbidities such as diabetes mellitus, cigarette smoking, CYP2C19 metabolizer genotype, and non-adherence to therapy; however, it remains the most significant [74,75,76,77]. Genotypic methods for detecting antibiotic resistance hold promise for identifying those at risk of treatment failure but are not yet sufficiently sensitive nor universally available. Local antibiotic resistance data are most useful in guiding the choice of empiric therapy but is not always current or reliably reported. Collection and dissemination of local surveillance data will be crucial to informing the choice of cost-effective empiric therapies and identifying whom to test for antibiotic resistance, and relevant stakeholders should work together to remove barriers to gathering and sharing such data. Assessment of a patient’s demographic and clinical characteristics associated with antibiotic resistance may be a helpful adjunctive tool in guiding the choice of empiric therapy. Identification of previous antibiotic exposures is likely to be most helpful in this regard, but other factors should be considered as patients are not always able to accurately recall past antibiotic use. Further study of these risk factors is warranted and should be evaluated as part of the much-needed surveillance studies of local *H. pylori* antibiotic resistance patterns. Better understanding of these factors may help clinicians choose the most appropriate and cost-effective empiric therapies for each patient. For example, patients with multiple clinical factors that predict antibiotic resistance may be better served with first-line bismuth-based quadruple therapy or high-dose dual therapies, which may be more effective against resistant strains [78]. Emerging treatments such as nitazoxanide, furazolidone, and vonoprazan may hold even more therapeutic potential in patients with high likelihood of antibiotic resistance [79,80,81,82,83,84]. Similarly, awareness of patients at greater risk for antibiotic resistant strains could help identify those that would be good candidates for susceptibility testing, particularly among those who have experienced treatment failure. Although universal, rapid, non-invasive susceptibility testing would be ideal prior to initiation of therapy, present limitations imposed by cost, access, and sensitivity highlight the potential utility of individual factors to inform who may benefit most from these tools. Such a strategy may improve eradication rates and minimize the costs of inappropriate antibiotic use.

## Data Availability

All data referenced in this review were reported in PubMed-indexed literature available online.
